# Discrete Choice Model of Food Store Trips Using National Household Food Acquisition and Purchase Survey (FoodAPS)

**DOI:** 10.3390/ijerph14101133

**Published:** 2017-09-27

**Authors:** Amy Hillier, Tony E. Smith, Eliza D. Whiteman, Benjamin W. Chrisinger

**Affiliations:** 1 School of Social Policy & Practice, University of Pennsylvania, Philadelphia, PA 19104, USA; 2 Department of Electrical and Systems Engineering, School of Engineering, University of Pennsylvania, Philadelphia, PA 19104, USA; tesmith@seas.upenn.edu; 3 Department of City and Regional Planning, School of Design, University of Pennsylvania, Philadelphia, PA 19104, USA; elizadw@design.upenn.edu; 4 Stanford Prevention Research Center, School of Medicine, Stanford University, Stanford, CA 94305, USA; chrisinger@stanford.edu

**Keywords:** FoodAPS, discrete choice, food shopping, supermarkets, food retail

## Abstract

Where households across income levels shop for food is of central concern within a growing body of research focused on where people live relative to where they shop, what they purchase and eat, and how those choices influence the risk of obesity and chronic disease. We analyzed data from the National Household Food Acquisition and Purchase Survey (FoodAPS) using a conditional logit model to determine where participants shop for food to be prepared and eaten at home and how individual and household characteristics of food shoppers interact with store characteristics and distance from home in determining store choice. Store size, whether or not it was a full-service supermarket, and the driving distance from home to the store constituted the three significant main effects on store choice. Overall, participants were more likely to choose larger stores, conventional supermarkets rather than super-centers and other types of stores, and stores closer to home. Interaction effects show that participants receiving Supplemental Nutrition Assistance Program (SNAP) were even more likely to choose larger stores. Hispanic participants were more likely than non-Hispanics to choose full-service supermarkets while White participants were more likely to travel further than non-Whites. This study demonstrates the value of explicitly spatial discrete choice models and provides evidence of national trends consistent with previous smaller, local studies.

## 1. Introduction

Where households shop for food, including how they travel, how far they travel, and what stores they choose, is a central concern of health researchers and policymakers, serving as a proxy for access to healthful foods, particularly among low-income and food insecure households. At the federal level, a number of policies and programs focus on the retail food environment, including the location of stores and the foods they stock. These include the Healthy Food Financing Initiative (HFFI), aimed at developing and equipping food stores with healthful foods, the SNAP and the Special Supplemental Feeding Program for Women Infants and Children (WIC), which both require stores to be certified to redeem benefits, healthy corner store initiatives, and SNAP matching programs at farmers’ market. These programs are based in part on a belief that the type of food stores where households shop influences the nutritional quality of the foods that they purchase, which in turn influences the foods that they consume, which in turn influences obesity and chronic disease risk. Previous studies provide some evidence to support this causal process linking food store choice to health behaviors and health outcomes, as described below.

Numerous studies have found a correlation between the local food environment and health outcomes [1,2,3,4,5], but far fewer have used individual-level food purchasing data to assess the relationship between store type and the nutritional quality of food purchases. Among those that have, there are considerable differences in measures of nutritional quality and spatial relationships between households and food stores as well as sample size and generalizability.

Two studies have used large nationally-representative surveys—Nielsen’s US Homescan Consumer Panel dataset from 2000–2012, which includes 670,782 household year observations [6], and the US Department of Agriculture’s Household Food Acquisition and Purchase Survey (FoodAPS) which includes detailed food shopping data for 4826 households over one week in 2012 or early 2013 [7].

The longitudinal study of the Nielsen Homescan data found that the proportion of packaged food purchases (PFP) made at mass merchandisers, convenience stores, and warehouses/clubs increased over time while the proportion of foods purchased at supermarkets (chain and non-chain) decreased. This change had implications for nutrition, as measured by food groups and nutrient profiles, as the PFPs that households made at mass merchandisers, convenience stores and warehouse/clubs had more energy, sugar, sodium, and saturated fat than foods purchased at supermarkets [6]. The study using FoodAPS focused exclusively on beverages, categorized as sugar-sweetened beverages or low-calorie beverages and water, and found higher odds of purchasing sugar-sweetened beverages at supercenters and convenience stores than at other types of stores [7].

The Nielsen Homescan study did not incorporate the location of households into the analysis. The FoodAPS study considered the availability of different types of food stores within a mile of the home of participants, as well as the distance from home to chosen food store. The study compared households receiving SNAP, household not receiving SNAP but income-eligible, and households not income-eligible for SNAP. SNAP households with a supermarket within a mile of their home were more likely to shop at a supermarket and less likely to shop at supercenters, while SNAP households with a supercenter within a mile of their home were more likely to shop at a supercenter and less likely to shop at a supermarket. Distance traveled from home to food store was used as a control variable and thus not the focus of the analysis [7].

Most of the studies that have investigated the relationship between food store choice and the nutritional quality of food purchases have not relied on large secondary data sets and thus used much smaller samples from a single geographic area, either intercept surveys outside specific food stores or surveys of household primary food shoppers about their food shopping choices. One study using intercept surveys outside of small and non-traditional food stores—corner stores, gas-marts, pharmacies and dollar stores—in Minneapolis/St. Paul, MN showed that few customers purchased fruits and vegetables or whole grains. The Healthy Eating Index (HEI-2010) scores of food purchases were associated with the amount of shelf space dedicated to fruits and vegetables and the ratio of shelf space devoted to healthy vs. less healthy items. The amount of added sugar in foods purchased at these stores far exceeded national dietary recommendations, with a mean HEI score of 36.4 (out of 100), and sugar-sweetened beverages were the most common purchases [8]. Separate analysis of a similar sample of intercept surveys in Minneapolis/St. Paul, MN found that a small proportion (11%) of customers purchased healthy foods and beverages and a majority (71%) purchased unhealthy foods [9].

A study based on intercept surveys outside four New York City bodegas found the most common food purchases to be sugary beverages, sugary snacks, coffee, sandwiches, and non-baked potato chips—unhealthy items, for the most part [10]. A study of 175 food shoppers in Baltimore using the consumer impact questionnaire to develop a healthy-food-getting score found that participants were more likely to purchase unhealthy food when walking to corner stores and more likely to purchase healthy food when driving to the food stores [11]. A study of 266 African-American women in Detroit used the fruit and vegetable module from the 2001 Behavioral Risk Factor Surveillance System to assess fruit and vegetable intake, linking more frequent consumption of fruit and vegetables to shopping more often at supermarkets and specialty stores than independent grocers [12]. Together, these studies consistently show more healthful food purchases at supermarkets and less healthful food purchases at smaller stores, particularly corner stores/bodegas. They provide somewhat less evidence that purchases at mass merchandizers, supercenters, and warehouses are less healthful, on the whole, than those from supermarkets.

A larger body of research about where people shop for food has established some clear travel and food choice patterns [13,14,15,16,17,18,19]. Most people do most of their food shopping at full-service supermarkets—even when none exist in their neighborhood—and drive or get a ride to do most of their food shopping. Also, most people do not shop at the closest food store, or even closest supermarket. Beyond these general patterns, individual and household characteristics, such as race/ethnicity, car ownership, and income, help explain some of the variability in store choice.

Our study builds on this growing body of research by examining the large, nationally-representative household sample in the USDA’s FoodAPS data set [20]. A previous analysis of the FoodAPS data found that households participating in SNAP as well as food-insecure households were less likely to drive their own car to do their primary food shopping and were more likely to get rides from someone else or seek other means of transportation such as walking, biking, or public transportation. However, SNAP and food-insecure households did not shop at different types of food stores from the rest of the population, and like other households, they frequently traveled beyond the closest food store when choosing where to shop [21]. That study compared shopping behavior between relevant pairs of consumer groups (such as SNAP and non-SNAP consumers), and used simple difference-between-means tests to compare group mean distances to both primary food stores and closest SNAP stores, as well as mean shares of chosen outlet types. Such pairwise comparisons cannot control for other relevant attributes of consumers, or for the availability of other shopping alternatives.

Our research extends the previous analysis of FoodAPS data by employing a spatially explicit discrete choice model that considers where the households shop relative to other households living near them as well as urban and rural differences. Closest to our present work is the study by Taylor and Villas-Boas [22], which also used the FoodAPS dataset and applied a discrete choice framework to assess how consumers shopping for both food consumed at home (FAH) and food consumed away from home (FAFH) choose among multiple food outlet alternatives. Their main results were that households will travel further to shop at superstores, supermarkets, and fast food outlets than at farmers’ markets and smaller grocery stores and that these differences vary across households by income and SNAP status. However, rather than focusing on individual store choices, this analysis aggregated stores into relevant outlet types and characterized “shopping choices” in terms of expenditure shares (relative to all other shopping expenditures). In this setting, the analysis reduced to a simple linear regression of (log) shares on individual and outlet attributes. In particular, the key spatial variable, shopping distance, was replaced (instrumented) by the closest-store distance within each outlet type. As discussed below, this largely ignores the relevant choice set of shopping alternatives actually available to consumers.

Hillier et al. [23] used a conditional logit model to assess how the characteristics of individuals and their households interact with food store characteristics, including distance from their home, in determining where to shop for food. Essential to such models is the identification of a choice set—the group of stores from which individuals are likely to choose. This methodological approach is intended to better approximate the real-life decision-making processes of households than approaches that consider endless alternatives. From a conceptual viewpoint, this type of discrete choice model focuses on how such attributes may influence probable store choices by individuals from sets of available alternatives. Choice sets were defined based on the food stores chosen by households living on the same residential block. Results highlighted the importance of distance from home to food stores, overall, but also demonstrated the influence on food store choice of the race and sex of food shoppers, travel mode, and where they spend time other than at home, as well as food prices and the availability of healthful foods. The main limitation of this study was the small sample (n = 467) from a set of contiguous neighborhoods in a single city, thus limiting generalizability. In the current study, we used the same discrete choice model employed in this previous study [23] with the much larger and more generalizable FoodAPS dataset to address the following research questions: (1) Where do participants shop for food at home (FAH); and (2) How do the individual and household characteristics of food shoppers interact with store characteristics and distance from home to store?

## 2. Materials and Methods

Through USDA’s FoodAPS initiative, data was collected about foods purchased or otherwise acquired from a nationally representative sample of 4826 households [20]. The location of residence, as measured by census block group, was recorded for each participating household. In addition to detailed data about the location of FAH and FAFH purchases, FoodAPS includes data about the retail food environment from Nielson TDLinx and the Store Tracking and Redemption System (STARS) maintained by the USDA’s Food and Nutrition Service.

### 2.1. Characteristics of Households, Food Shoppers and Food Stores

The primary food store identified by the primary food shopper for each participating household served as the relevant choice variable, while the characteristics of stores served as explanatory variables. Type of store was measured using the TDLinx sub-type or sub-channel: conventional supermarket, supercenter, limited assortment, conventional club, natural/gourmet food, dollar store, conventional mass merchandizer, conventional convenience, warehouse grocery, military convenience, and military commissary. We converted this to a dummy variable indicating conventional supermarket or not (**SUPMKT)**. Store size was measured in square-footage (**SQFT**).

Certain characteristics of the primary food shopper and their household were also employed as explanatory variables. These included sex (**SEX**; female or not), race (**RACE**; White or not), ethnicity (**HISP**; Hispanic or not); SNAP participation (**SNAP**), car ownership (**CAR**), and driving distance to primary store from home (**DIST**) from the individual and household. We also included the percent urban population of the county in which the participant lived (**URBAN**; measured continuously first, then dichotomized at 90%) from the 2010 US Census to better understand urban/rural, particularly in regard to distance traveled to primary food store.

### 2.2. Conditional Logit Model

Consistent with our approach in Hillier et al., 2015 [23], we used a conditional logit model to determine how individual shopper, trip distance, and food store characteristics interact and help explain food store choice. Given a set of *individuals* (*households*) i∈I and *stores*, s∈S, if the choice set of store alternatives relevant for individual, i, is denoted by $S_{i}\subseteq S$, then our *conditional logit model* takes the general form

(1) $$P_{i}(s)=\frac{\exp(V_{is})}{\sum_{s^{\prime }\in S_{i}}\exp(V_{is^{\prime }})},\;s\in S_{i},\;i\in I$$

where $P_{i}(s)$ denotes the probability that store s is chosen by individual i from set $S_{i}$. These choice probabilities are assumed to depend on the *value*, $V_{is}$, of each store s to individual i. As in linear regression, these values are assumed to be representable as linear functions of a relevant set of store attributes, $\left(x_{sj}:j=1,..,J\right)$, such as the size and availability of healthful foods at store s. These values may differ among individuals, depending on attributes, $\left(z_{ik}:k=1,..,K\right)$, such as the sex and race of the individual. Such value differences can be captured by interacting individual attributes with each store attribute. As with store attributes, the value of distance accessibility may differ among individuals. For example, distance may be less important for car owners. Such effects can again be captured by interacting these distances with individual attributes. Hence in the most general model considered here, values of stores for individuals are taken to be linear functions of the form:

(2) $$V_{is}=\sum_{j=1}^{J}\left[\beta_{j}x_{sj}+\sum_{k=1}^{K}\beta_{kj}z_{ik}x_{sj}\right]+\sum_{h=1}^{2}\left[\theta_{h}d_{h}(is)+\sum_{k=1}^{K}\theta_{kh}z_{ik}d_{h}(is)\right]$$

where the first term on the right-hand side involves store attributes together with individual interaction effects and the second term involves distance together with their individual interaction effects.

Following standard terminology, coefficients $\beta_{j}$ and $\theta_{h}$ are referred to as the “main effects” for store attribute j and distance attribute h, respectively. Similarly, for any given individual attribute, k, coefficients $\beta_{kj}$ and $\theta_{kh}$ are referred to as “interaction effects” between k and, respectively, store attribute, j, and distance attribute, h. To interpret these coefficients, note for example that the effects of store attribute j can be isolated by considering two hypothetical stores, s and $s^{\prime }$, that differ only with respect to attribute j. To capture the effects of a unit change in attribute, j, suppose in addition that $x_{sj}-x_{s^{\prime }j}=1$. Then the relative likelihood of any individual i choosing store s versus $s^{\prime }$ is seen from (1) and (2) to be of the form:

(3) $$P_{i}(s)/P_{i}(s^{\prime })=\exp\left[\beta_{j}(x_{sj}-x_{s^{\prime }j})+\sum_{k=1}^{K}\beta_{kj}z_{ik}(x_{sj}-x_{s^{\prime }j})\right]=\exp\left(\beta_{j}+\sum_{k=1}^{K}\beta_{kj}z_{ik}\right)$$

Thus, in this context it is clear that the “main effect”, $\beta_{j}$, reflects that component of change in the relative likelihood of choosing s versus $s^{\prime }$ which is common to *all* individuals, i. (Technically one should add “for all individuals for whom both s and $s^{\prime }$ are relevant options”. But since $\beta_{j}$ is clearly independent of these particular option choices, we ignore this complication.) Similarly, $\beta_{kj}$, reflects the additional component of change in this relative likelihood that is specific to individuals with $k^{th}$ attribute level, $z_{ik}$. (By taking the logs in (3), these can also be interpreted as linear changes in “log odds”. Alternatively, one can obtain interpretations in terms of “elasticities” and “cross-elasticities” of substitution, as for example in Section 3.6 of Train (2009).) Parallel interpretations can be given to the distance parameters, $\theta_{h}$ and $\theta_{kh}$. We ran a single conditional model that included the main and interaction effects.

### 2.3. Store Choices and Choice Sets

**Figure 1:**
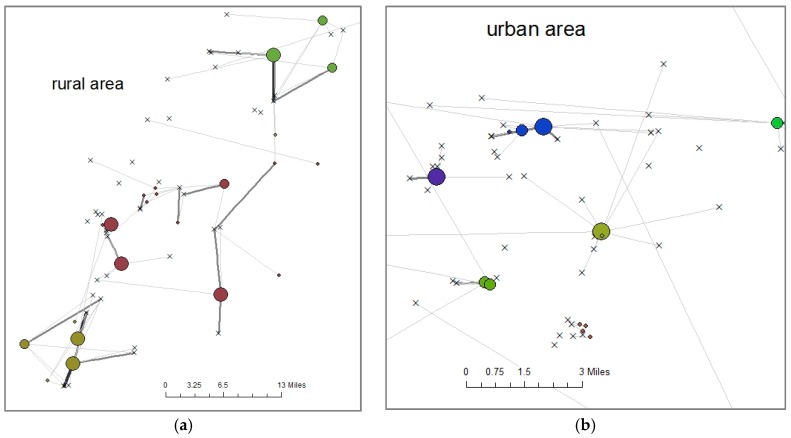
These figures show food shopping clusters where the small x’s represent food stores, small black dots represent the centroid of block groups where participants live and the colored circles represent food stores chosen, where larger circles indicate that more households chose that particular store and each different color indicates a distinct shopping cluster. Where x’s are connected to colored circles, one or more participants chose that store as their primary food store while x’s with no lines connecting them to colored circles represent food stores that were not chosen as the primary food store for any participants. (**a**) This figure shows three food shopping clusters within a rural area where travel distances to food stores were typically greater; (**b**) This figure shows two shopping clusters, and part of a third, in an urban area where residents have many more store choices within a smaller geographic area. Note the different geographic scales of the two maps.

We approached the question of choice set—the pool of stores from which individual shoppers are choosing—differently from Hillier et al., 2015. We defined the relevant store choice for each individual i to be the primary food store used by the primary adult respondent in the FoodAPS household and the relevant choice set, $S_{i}$, for each individual i to be the set of all store choices made by individuals in i’s shopping cluster (For additional discussion of such choice-set identification issues see, for example, Fotheringham (1988) and Pelligrini (1997).) We created these shopping clusters by grouping nearby block groups where participants lived using visual inspection of maps in ArcGIS showing lines between block group centroids and the primary food stores chosen by participants in each block group. Each block group could only be in one shopping cluster. This approach generated 221 shopping clusters that included a maximum of 105 different participants and 18 different stores, a minimum of one participant and one store, with a median of 13 participants and five stores. These areas ranged widely in size. The cumulative area was calculated based on the block groups in which participants lived within the same shopping cluster. For 93 of the 221 clusters—in highly urbanized areas—this cumulative area was less than a square mile. The largest area, in a rural section of the Midwest, had a cumulative area of 660 square miles. The average area was 30.7 square miles and the median was 2.6 square miles. See Figure 1.

## 3. Results

Only primary shoppers for whom characteristics were known about their primary food store were included in the analyses. Data on store characteristics were incomplete for 693 of the primary stores chosen, leading to a sample of 4015 households (reduced from 4826). Stores that are not authorized to accept SNAP benefits made up a portion of the stores with missing data. As a result, all of the primary stores chosen and included in our analyses were authorized to accept SNAP benefits. We further eliminated participants choosing stores too far to be relevant choices for others in their shopping cluster. We did this manually by visually inspecting all participant–primary-food-store combinations in ArcMap that involved a distance of 10 miles or more. This led us to develop the rule that if a store trip was more than twice as long as the next longest trip in the shopping cluster, we would eliminate it. This led to the removal of an additional 18 households and a final sample of 3997. Upon further inspection, the primary food shopper was working outside the home in 12 of these 18 households and in five of those 12 households, the primary food shopper had a commute of 45 min, two had commutes of 30 min, and one had a commute of 20 min. These relatively long commutes might help explain these outliers. The sample was made up predominantly of women, Whites, and people with access to a car (See Table 1).

The 3997 households chose 1104 unique stores as their primary food store. The majority (59.1%) of these were conventional supermarkets. Supercenters, such as Wal-MART and Super Target, made up the next largest segment (15.3% of stores), followed by limited assortment stores (9.6%), wholesale clubs (6.2%), and natural/gourmet stores (4.1%). The majority of households chose conventional supermarkets as their primary store (61.1%). Another 25.1% chose supercenters, 6.5% chose limited assortment stores, 2.7% chose wholesale clubs, and 1.7% chose natural/gourmet stores.

**SQFT**, **SUPMKT** and **DIST** were the three significant main effects in the conditional logit model. Overall, participants were more likely to choose larger stores, conventional supermarkets rather than other types of food stores, and stores closer to home. Interaction effects show that participants receiving SNAP were even more likely to choose larger stores (**SQFT-SNAP**) while participants in highly urbanized areas were less likely to choose larger stores than their rural counterparts (**SQFT-URBAN**). Hispanic participants were more likely than non-Hispanic participants to choose full-service supermarkets (**SUPMKT-HISP**). White participants were more likely to travel further than non-White participants (**DIST-RACE**), as were participants who owned a car (**DIST-CAR**) and participants living in less urbanized areas (**DIST-URBAN**).

Because SNAP status was not significant in the conditional logit model, we looked more closely at the relationship between the receipt of SNAP benefits and store choice. Pairwise correlations showed that SNAP was most strongly (negatively) related to RACE. As an experiment, we dropped RACE from the conditional logit model to see if there was an effect on SNAP, and only SQFT-SNAP increased in significance. Finally we removed HISP and SEX as well, again the conclusion was the same. As one last check, we removed SNAP altogether and found that DIST-RACE and DIST-CAR were slightly more significant but with no real qualitative changes. Finally, we considered other attributes in the same way. By dropping SEX, SQFT-SNAP and DIST-CAR interactions became more significant, but there were no qualitative changes. Similarly, dropping HISP or RACE had no qualitative effects, so the conditional logit results presented in Table 2 were adopted as final.

## 4. Discussion

This study builds on previous research linking food store choice to the nutritional quality of food purchases by utilizing an explicitly spatial discrete choice model to analyze a large national dataset. Our results are largely consistent with previous research. All things being equal, the primary food shopper chose larger supermarkets closer to home. Of course, all things are not equal and these results show meaningful differences in food store choice across sex, race, ethnicity, car ownership, and rural/urban locations.

While SNAP was not significant, race/ethnicity was. Ethnicity (Hispanic or not) had a significant interaction effect on the choice of food store type (supermarket or not), and race (White or not) had a marginally significant interaction effect on distance traveled. While it is not immediately apparent what impact this might have for racial/ethnic health disparities over the long term, these findings indicate that race/ethnicity may have a stronger influence over where people shop than income, as measured by SNAP. Previous research has demonstrated considerable racial/ethnic sorting when it comes to food shopping [24], which likely reinforces and even increases racial/ethnical segregation within household food store choice. Qualitative studies have shown differences in the food shopping experiences of Black/African-American and Latina women, relative to other women, reflecting different physical food environments, experiences with discriminatory treatment, family preferences and perceptions of value [25,26,27,28].

That SNAP status did not interact significantly with store type or distance traveled, even when other food shopper and household characteristics were removed from the model, suggests that SNAP households make choices about their primary food store based largely on the same factors as households that do not receive SNAP. More detailed analysis of FAH purchases that consider the type of payment—specifically whether SNAP benefits are used during a particular transaction—and time of month relative to distribution of SNAP benefits might reveal some differences in food store choice between SNAP and non-SNAP households when SNAP benefits are used.

### 4.1. Limitations

This study has a number of limitations. We only looked at the primary food store identified by the self-identified primary food shopper for the household, but most households shop at multiple stores, and multiple household members may make FAH purchases. The FoodAPS dataset does include information about these other food trips, but discrete choice analyses would likely not work with this number of possible choices. Our definition of choice sets offered an improvement over previous research, but it still did not meet the ideal of including all of the store-choice options actually perceived to be relevant by individuals. The conditional logit model offers an improvement over random choice, but it still has only a 36% success rate, meaning that much of the variability in store choice is not explained by the variables in our model. We had only a limited number of food store characteristics to include. Ideally, we would have also included variables about food prices and selection as well as perceptions of customer service and safety. Finally, distance was measured from home to the primary food store even though food store trips may take place near work or other places where people spend time or along paths they travel between such locations.

### 4.2. Strengths

Most of these limitations reflect the limits of the FoodAPS dataset, but the availability of FoodAPS allowed for the use of a large national sample, which is a primary strength of this study. Our statistical model accounts for spatial patterns in food shopping, through driving distance to the primary food store and identification of food shopping clusters based on where neighboring households shop. Finally, the discrete choice model used in this study focused on individual store-choice events, thus allowing consumer, store and distance attributes to be captured explicitly for both the store chosen and all other relevant store choices.

## 5. Conclusions

Understanding how people make food purchasing decisions is critical to understanding how and where to intervene in order to influence health outcomes, by changing behavior, changing the food environments, or both. These results indicate that SNAP status may not be as important as race/ethnicity in understanding food store choices. Successful interventions aimed at influencing household shopping behavior must recognize—if not reinforce—these distinctions.

The food environment has become increasingly complicated, with a wide range of food retail options including limited assortment and discount stores, large chain convenience stores, pharmacies, and supercenters competing with supermarkets for market share. Even within the same general type of stores, significant variability exists in the availability of healthful foods. Despite all the options, conventional supermarkets remain the most popular choice across SNAP status, although there are meaningful differences based on car ownership, race/ethnicity, and distance from home to store. The evidence is still limited, but there are indications that people buy different types of foods at different types of stores. Public policy needs to focus on ensuring that everyone across race, ethnicity, and income has food outlets with healthful options within a manageable travel distance, in-store environments—including marketing, pricing, and shelf-space—that promote healthful choices, and the financial ability to choose healthful items.

## Figures and Tables

**Table 1:** Characteristics of households, primary food shoppers, and residential location.

**Variable**	**Count (%)**			
Sex (female)	2942 (73.6%)			
Race (White)	2863 (71.6%)			
Hispanic (yes/no)	786 (19.7%)			
Car access (yes/no)	3387 (84.7%)			
SNAP participation	1293 (32.2%)			
**Variable**	**Min**	**Max**	**Median**	**Std Dev**
Store size (square feet)	1000	185,000	44,000	25,660
Distance to store (miles)	0.03	73.95	2.31	5.43
urbanized (%)	0	100	88	26.4

**Table 2:** Conditional logit model results.

Variable	Parameter	z-Value ^†^	Probability
SQFT	0.0170	6.6443 ***	0.0000
SQFT-RACE	0.0020	1.0386	0.2990
SQFT-HISP	0.0012	0.6337	0.5263
SQFT-SNAP	−0.0028	−1.9098 *	0.0562
SQFT-CAR	−0.0017	−1.1142	0.2652
SQFT-SEX	−0.0016	−1.0648	0.2869
SQFT-URBAN	−0.0072	−4.9555 ***	0.0001
SUPMKT	0.0169	2.5370 **	0.0112
SUPMKT-RACE	−0.0038	−0.7551	0.4502
SUPMKT-HISP	0.0114	2.4424 *	0.0146
SUPMKT-SNAP	−0.0027	−0.7049	0.4809
SUPMKT-CAR	0.0013	0.3189	0.7498
SUPMKT-SEX	−0.0017	−0.4603	0.6453
SUPMKT-URBAN	−0.0049	−1.2892	0.1973
DIST	−0.3736	−8.6711 ***	0.0000
DIST-RACE	0.0631	1.8772 *	0.0605
DIST-HISP	0.0105	0.3505	0.7259
DIST-SNAP	−0.0043	−0.2071	0.8359
DIST-CAR	−0.0043	1.9626 **	0.0497
DIST-SEX	0.0368	1.6955 *	0.0900
DIST-URBAN	−0.1745	−7.4888 ***	0.0000
Success rate	38.03%		
Model success rate	25.63%		
Random success rate	18.26%		

**^†^** *** = |*p*| < 0.01, ** = |*p*| < 0.05, * = |*p*| < 0.10.
